# A Prospective Study Evaluating Metabolic Capacity of Thiopurine and Associated Adverse Reactions in Japanese Patients with Inflammatory Bowel Disease (IBD)

**DOI:** 10.1371/journal.pone.0137798

**Published:** 2015-09-11

**Authors:** Shunichi Odahara, Kan Uchiyama, Takahiro Kubota, Zensho Ito, Shinichiro Takami, Hiroko Kobayashi, Keisuke Saito, Shigeo Koido, Toshifumi Ohkusa

**Affiliations:** 1 Department of Internal Medicine, Division of Gastroenterology and Hepatology, The Jikei University School of Medicine (Kashiwa Hospital), Chiba, Japan; 2 Department of Biopharmaceutics, Faculty of Pharmaceutical Sciences, Niigata University of Pharmacy and Applied Life Sciences, Niigata, Japan; Cincinnati Children's Hospital Medical Center, University of Cincinnati College of Medicine, UNITED STATES

## Abstract

Azathioprine (AZA) is frequently used in patients with inflammatory bowel disease (IBD). However, toxic adverse reactions frequently develop and limit the clinical benefits. Currently, the precise mechanisms underlying thiopurine-related toxicity are not well understood.

To investigate the relationship between the extent of thiopurine metabolism and adverse reactions in Japanese IBD patients, we prospectively observed 48 IBD patients who received AZA. We analyzed the thiopurine *S*-methyltransferase (TPMT) and inosine triphosphate pyrophosphatase (ITPA) gene mutations and measured the concentrations of 6-thioguanine nucleotide (6-TGN) continuously for 52 weeks. All patients possessed wild-type TPMT gene sequences. The ITPA *94C>A* mutation was detected in 19 patients (39.6%). Adverse reactions developed in 14 of the 48 patients (29.2%), including leukopenia in 10 patients (20.8%). In the leukopenia group, the percentages of patients with *94C>A* were higher than those in the without-leukopenia group (70.0% vs. 31.6%, *P* < 0.05). The average concentrations of 6-TGN in the patients with *94C>A* were generally higher than those in the patients without *94C>A*, however, there were no significant differences. Only 3 out of 10 patients with leukopenia exhibited high 6-TGN levels (30.0%). No negative correlations between white blood cell (WBC) counts and 6-TGN concentrations were observed. The cumulative incidence of leukopenia were higher for patients with *94C>A*. Seven out of 19 patients (36.8%) with the ITPA *94C>A* mutation developed leukopenia; however, this mutation may not unequivocally increase the risk of developing leukopenia. In addition, there are factors other than increased 6-TGN levels that are involved in the onset of leukopenia.

## Introduction

Azathioprine (AZA) is frequently used for steroid discontinuation and remission maintenance in patients with inflammatory bowel disease (IBD). However, toxic adverse reactions, including myelosuppression, frequently develop and limit the clinical benefits of this drug. The adverse reactions are divided into type A (those caused by the dose-dependent pharmacological activity of AZA/6-mercaptopurine (6-MP)) and type B (those involving allergic reactions and lacking dose dependency). The type A adverse reactions include myelosuppression (such as leukopenia and thrombocytopenia), alopecia, and increased susceptibility to infection and hepatitis, whereas type B reactions include fever, eruptions, arthralgia, myalgia, gastrointestinal symptoms (such as nausea), malaise and pancreatitis [1]. Currently, the precise mechanisms underlying thiopurine-related toxicity are not well understood. We aimed to elucidate thiopurine metabolism and its impact on patient toxicity.

**Fig 1:**
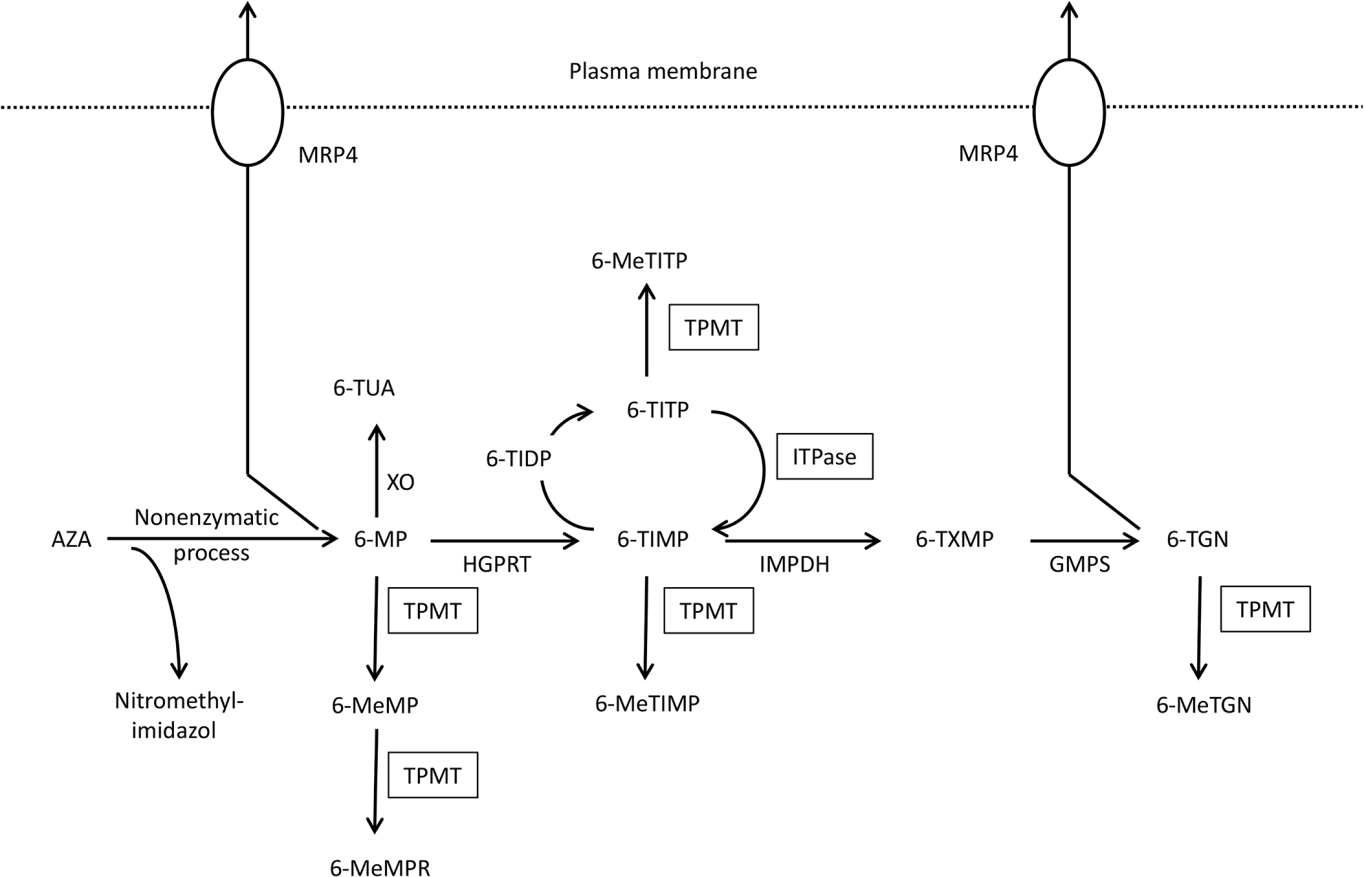
Metabolism and transportation of AZA/6MP and its metabolites. XO, xanthine oxidase; TPMT, thiopurine S-methyltransferase; HGPRT, hypoxanthine–guanine phosphoribosyl transferase; ITPA, inosine triphosphate pyrophosphatase; IMPDH, inosine monophosphate dehydrogenase; GMPS, guanosine monophosphate synthetase; AZA, azathioprine; 6-MP, 6-mercaptopurine; 6-TUA, 6-thiouric acid; 6-MeMP, 6-methylmercaptopurine; 6-MeMPR, 6-methylmercaptopurine ribonucleotide; 6-TIMP, 6-thioinosine monophosphate; 6-TIDP, 6-thioinosine diphosphate; 6-TITP, 6-thioinosine triphosphate; 6-MeTIMP, 6-methylthioinosine monophosphate; 6-MeTITP, 6-methylthioinosine triphosphate; 6-TXMP, 6-thioxanthosine 5’-monophosphate; 6-TGN, 6-thioguanine nucleotide; 6-MeTGN, 6-methylthioguanine nucleotide.

Upon oral administration, AZA is absorbed into the plasma and is converted to 6-MP in a nonenzymatic reaction occurring within erythrocytes. Three major pathways then convert 6-MP into its various metabolites. The three critical enzymes corresponding to these pathways are xanthine oxidase (XO), thiopurine *S*-methyltransferase (TPMT), and hypoxanthine guanine phosphoribosyl transferase (HGPRT). The activity of TPMT is critically important in determining the balance between the production of 6-thioguanine nucleotide (6-TGN) and that of 6-methylmercaptopurine ribonucleotide (6-MeMPR). Individuals with lower TPMT activity display higher 6-TGN concentrations. Inosine triphosphate pyrophosphatase (ITPase) converts 6-thioinosine triphosphate (6-TITP) back to 6-thioinosine monophosphate (6-TIMP) so that 6-TITP does not accumulate. A deficiency in ITPase interrupts this cycle, leading to the accumulation 6-TITP (Fig 1). We have previously reported that the inosine triphosphate pyrophosphatase (ITPA) gene mutation is closely related to adverse AZA/6-MP reactions in Japanese patients [2]. This prospective study was performed to investigate the relationship between the extent of thiopurine metabolism and adverse toxic reactions in thiopurine-naïve Japanese IBD patients. This study is the first investigation in which blood concentrations of 6-TGN have been continuously monitored over a long-term period in IBD patients being treated with AZA.

## Materials and Methods

### Subjects

This study analyzed mutations in the TPMT and ITPA genes and the levels of specific metabolic products in IBD patients. From March 2008 to June 2011, we prospectively observed 50 Japanese IBD patients for 52 weeks who received AZA. Whole blood samples were drawn at 0, 1, and 2 weeks and then every 4 weeks. We analyzed the TPMT and ITPA gene mutations and measured the concentrations of 6-TGN after observation or after interruption due to adverse reactions. In Western countries, an AZA dosage of 2–3 mg/kg/day is recommended for the treatment of IBD patients [3], but lower dosages (0.6–1.2 mg/kg/day) are used in Japanese individuals because of their relatively heightened sensitivities [4]. In this study, the initial AZA dosage was 1.0 mg/kg/day.

### TPMT, ITPA

For the TPMT gene analysis, the mutant alleles that reportedly reduce enzyme activity, i.e., *3C and *6, were analyzed by PCR-RFLP, and the other mutations by sequence analysis [2,5]. For the ITPA gene analysis, we screened for the *94C>A* mutation, which causes reduced ITPA activity.

### 6-TGN

The 6-TGN levels in the red blood cells (RBCs) were measured using high-performance liquid chromatography (HPLC) as described by Lennard and Maddocks [6] with minor modifications [7]. The blood samples were placed in EDTA-2K tubes and stored at -20°C before further processing. Then, 500 μL of blood, 50 μL 100 mg/mL dithiothreitol, 50 μL 70% perchloric acid, and 500 μL dichloromethane were added to a new tube. After vortexing for 30 seconds, the tube was centrifuged for 15 minutes at 13,000 rpm, and then, 450 μL of the supernatant was transferred to another tube and hydrolyzed for 120 minutes at 105°C. The mixture was cooled to room temperature, and an additional centrifugation was performed for 3 minutes at 3,000 rpm. Next, 100 μL 2 M NaOH was added, and after vortexing for 30 seconds, the mixture was centrifuged for 5 minutes at 13,000 rpm. Then, 25 μL of the supernatant as analyzed at a flow rate of 500 μL/min on an Inertsil ODS-3 column (3.0 φ x 100 mm, 4 μm, GL Sciences, Tokyo, Japan) with an in-line filter (0.5-μm depth filter × 0.004 ID, Phenomenex, CA) at 50°C and a mobile phase of 40 mM KH_2_PO_4_ and 7 mM sodium 1-octanesulfonate, which was adjusted to pH 3.5, to which methanol (98/2 v/v) was added. Absorbance was detected at 340 nm. HPLC was performed using the LaChrom system (Hitachi, Tokyo, Japan). The inter-assay and intra-assay coefficients of variation for 6-TGN in the blood were less than 6.2%.

### Statistical analysis

In this study, leukopenia was defined as a white blood cell (WBC) count of 2500/μL or less. Hepatitis was defined as elevated serum alanine aminotransferase (ALT) and aspartate aminotransferase (AST) levels to over the upper limit of the normal range. For the analysis of adverse reactions, we considered the 6-TGN concentration at the time of diagnostic confirmation to be an adverse reaction, and for the analysis of the other factors, we also assessed the average 6-TGN concentrations from weeks 8 to 52 because these levels have been reported to remain largely consistent beyond week 4 [8].

All statistical analyses were performed using IBM SPSS Statistics version 22.

### Ethical considerations

These experiments were approved by the Ethics Committee of the Jikei University School of Medicine. We explained the purpose of the study and the methods involved to all participants prior to enrollment, and each individual provided their consent. Written informed consent was obtained from each participant after a full explanation of this study.

## Results

### Characteristics

Forty-eight of the 50 patients were observed for the complete duration of the study, and 2 patients dropped out. The characteristics of the patients are provided in Table 1. In total, 48 patients with a mean age of 34.2, consisting of 29 males (60.4%) and 19 females (39.6%), were included; 29 patients had ulcerative colitis (UC) (60.4%), and 19 had Crohn’s disease (CD) (39.6%).

**Table 1:** Baseline Patient Characteristics.

Total Patients	48
Age (years)	34.2 ± 13.6
Male: Female	29:19
Height (cm)	166.0 ± 8.3
Weight (kg)	54.6 ± 9.1
UC:CD	29:19
5ASA (+:-)	45:3
IFX (+:-)	17:31
ARs (+:-)	14:34
Leukopenia (+:-)	10:38

UC, ulcerative colitis; CD, Crohn’s Disease; 5ASA, 5-aminosalicylic acid; IFX, infliximab

ARs, adverse reactions. Mean ± standard deviation

### TPMT, ITPA

All patients possessed wild-type TPMT gene sequences. The ITPA *94C>A* mutation was detected in 19 patients (39.6%, all heterozygous, allele frequency: 19.8%).

### Adverse reactions

Adverse reactions developed in 14 of the 48 patients (29.2%), which involved some overlap, including leukopenia in 10 patients (20.8%), alopecia in 3 patients (6.3%), agranulocytosis in 1 patient (2.1%), hepatitis in 1 patient (2.1%), and rash in 1 patient (2.1%). One leukopenia patient developed agranulocytosis and alopecia. A total of six patients were discontinued AZA therapy due to adverse reactions (Table 2). The mean age of these patients was 39.8, and there were 4 males (28.6%) and 10 females (71.4%); 10 had UC (71.4%), and 4 had CD (28.6%). Onset was reported to occur from 7 to 364 days after AZA administration.

**Table 2:** Patients with Adverse Reactions during AZA therapy for IBD.

No.	Age	Sex	Disease	Adverse Reactions	TPMT mutation	ITPA mutation	Duration (days)	6-TGN (pmol/8×10^8^ RBCs)	Continuation of treatment
1	38	F	UC	Leukopenia	-	*94C/A*	116	327	Continuation*
2	55	M	UC	Leukopeina	-	*94C/A*	117	884	Continuation**
3	24	M	CD	Leukopeina	-	*94C/A*	7	219	Continuation***
4	66	F	UC	Leukopeina	-	-	40	347	Discontinuation
5	37	F	UC	Leukopeina	-	-	135	211	Continuation***
6	23	F	CD	Leukopeina	-	-	81	309	Continuation*
7	22	F	CD	Leukopeina	-	*94C/A*	91	733	Discontinuation
8	58	M	UC	Leukopeina	-	*94C/A*	15	95	Discontinuation
9	48	F	UC	Leukopeina	-	*94C/A*	364	814	Continuation**
10	31	F	UC	Alopecia	-	*94C/A*	59	327	Continuation**
11	16	F	UC	Agranulocytosis, Alopecia	-	*94C/A*	21	111	Discontinuation
12	50	F	UC	Alopecia	-	-	21	144	Discontinuation
13	65	F	UC	Hepatitis	-	-	60	479	Discontinuation
14	24	M	CD	Rash	-	-	147	113	Continuation*

AZA, azathioprine; IBD, inflammatory bowel disease; M, male; F, female; UC, ulcerative colitis; CD, Crohn’s Disease

TPMT, Thiopurine *S*-methyltransferase; ITPA, Inosine triphosphate pyrophosphatase; 6-TGN, 6-thioguanine nucleotide.

Continuation*, temporarily discontinuation; Continuation**, reduse dose; Continuation***, same dose.

### Retrospective evaluations

The comparisons of adverse reactions are presented in Table 3. The percentage of women in the adverse reactions group was higher than that in the group without adverse reactions (*P* < 0.02). Ten out of 19 women (52.6%) reported some adverse reactions.

**Table 3:** Characteristics of Adverse Reactions.

	Adverse reactions (ARs)	Without ARs	*P* value
	n = 14 (29.2%)	n = 34 (70.8%)	
Age (years)	39.8 ± 17.1	31.9 ± 11.6	N.S. *
Male:Female	4:10	25:9	*P* < 0.02 **
Height (cm)	162.3 ± 9.3	167.5 ± 7.6	N.S. ***
Weight (kg)	54.4 ± 11.1	54.8 ± 8.5	N.S. ***
UC:CD	10:4	19:15	N.S. **
5ASA (+:-)	14:0	31:3	N.S. **
IFX (+:-)	4:10	13:21	N.S. **
Mutation of TPMT (+:-)	0:14	0:34	N.S. **
Mutation of ITPA (+:-)	8:6	11:23	N.S. **
6-TGN (pmol/8×10^8^ RBCs)	365.2 ± 266.1	379.8 ± 177.1	N.S. ***
6-TGN >450:<450	4:10	10:24	N.S. **

UC, ulcerative colitis; CD, Crohn’s Disease; 5ASA, 5-aminosalicylic acid; IFX, infliximab

TPMT, Thiopurine *S*-methyltransferase; ITPA, Inosine triphosphate pyrophosphatase

6-TGN, 6-thioguanine nucleotide. N.S., not significant.

Mean ± standard deviation

* Mann-Whitney’s U-test

** Chi-square test

*** t-test.

The comparison involving leukopenia is presented in Table 4. In the leukopenia group, the percentages of woman and the rate of ITPA *94C>A* mutation were higher than those in the without-leukopenia group (*P* < 0.05). Seven out of 19 women (36.8%) in addition to 7 out of 19 of patients with *94C>A* (36.8%) had leukopenia. Three of the 14 patients (21.4%) whose average 6-TGN concentrations were over 450 pmol/8×10^8^ RBCs had leukopenia, and 7 of the 34 patients (20.6%) whose average 6-TGN concentrations were less than 450 pmol/8×10^8^ RBCs had leukopenia. There were no statistically significant differences between the groups with average 6-TGN concentrations of greater than and less than 450 pmol/8×10^8^ RBCs from 8 to 52 weeks.

**Table 4:** Characteristics of Leukopenia.

	Leukopenia	Without Leukopenia	*P* value
	n = 10 (20.8%)	n = 38 (79.2%)	
Age (years)	38.7 ± 17.4	33.0 ± 12.6	N.S. *
Male:Female	3:7	26:12	*P* < 0.05 **
Height (cm)	163.7 ± 9.9	166.6 ± 8.0	N.S. ***
Weight (kg)	54.2 ± 8.4	54.8 ± 9.5	N.S. ***
UC:CD	7:3	22:16	N.S. **
5ASA (+:-)	10:0	35:3	N.S. **
IFX (+:-)	3:7	14:24	N.S. **
Mutation of TPMT (+:-)	0:10	0:38	N.S. **
Mutation of ITPA (+:-)	7:3	12:26	*P* < 0.05 **
6-TGN (pmol/8×10^8^ RBCs)	405.0 ± 294.0	367.8 ± 177.7	N.S. ***
6-TGN >450:<450	3:7	11:27	N.S. **

UC, ulcerative colitis; CD, Crohn’s Disease; 5ASA, 5-aminosalicylic acid; IFX, infliximab

TPMT, Thiopurine *S*-methyltransferase; ITPA, Inosine triphosphate pyrophosphatase

6-TGN, 6-thioguanine nucleotide. N.S., not significant.

Mean ± standard deviation

* Mann-Whitney’s U-test

** Chi-square test

*** t-test.

The comparison of the patients with and without *94C>A* is shown in Table 5. The probability of leukopenia in the *94C>A* group was higher than that in the group without *94C>A* (36.8% vs. 10.3%, *P* < 0.05).

**Table 5:** Characteristics of patients with *94C/A* ITPA mutation.

	*94C>A*	Without *94C>A*	*P* value
	n = 19 (39.6%)	n = 29 (60.4%)	
Age (years)	35.0 ± 12.3	33.6 ± 14.8	N.S. *
Male:Female	11:8	18:11	N.S. **
Height (cm)	165.6 ± 9.4	166.3 ± 7.9	N.S. ***
Weight (kg)	52.8 ± 8.5	55.9 ± 9.6	N.S. ***
UC:CD	10:9	19:10	N.S. **
5ASA (+:-)	18:1	27:2	N.S. **
IFX (+:-)	6:13	11:18	N.S. **
ARs (+:-)	8:11	6:23	N.S. **
Leukopenia (+:-)	7:12	3:26	*P* < 0.05 **
6-TGN (pmol/8×10^8^ RBCs)	419.0 ± 220.8	333.7 ± 157.4	N.S. ***

UC, ulcerative colitis; CD, Crohn’s Disease; 5ASA, 5-aminosalicylic acid; IFX, infliximab

TPMT, Thiopurine *S*-methyltransferase; ITPA, Inosine triphosphate pyrophosphatase

6-TGN, 6-thioguanine nucleotide. N.S., not significant.

Mean ± standard deviation

* Mann-Whitney’s U-test

** Chi-square test

*** t-test.

### 6-TGN concentrations

**Fig 2:**
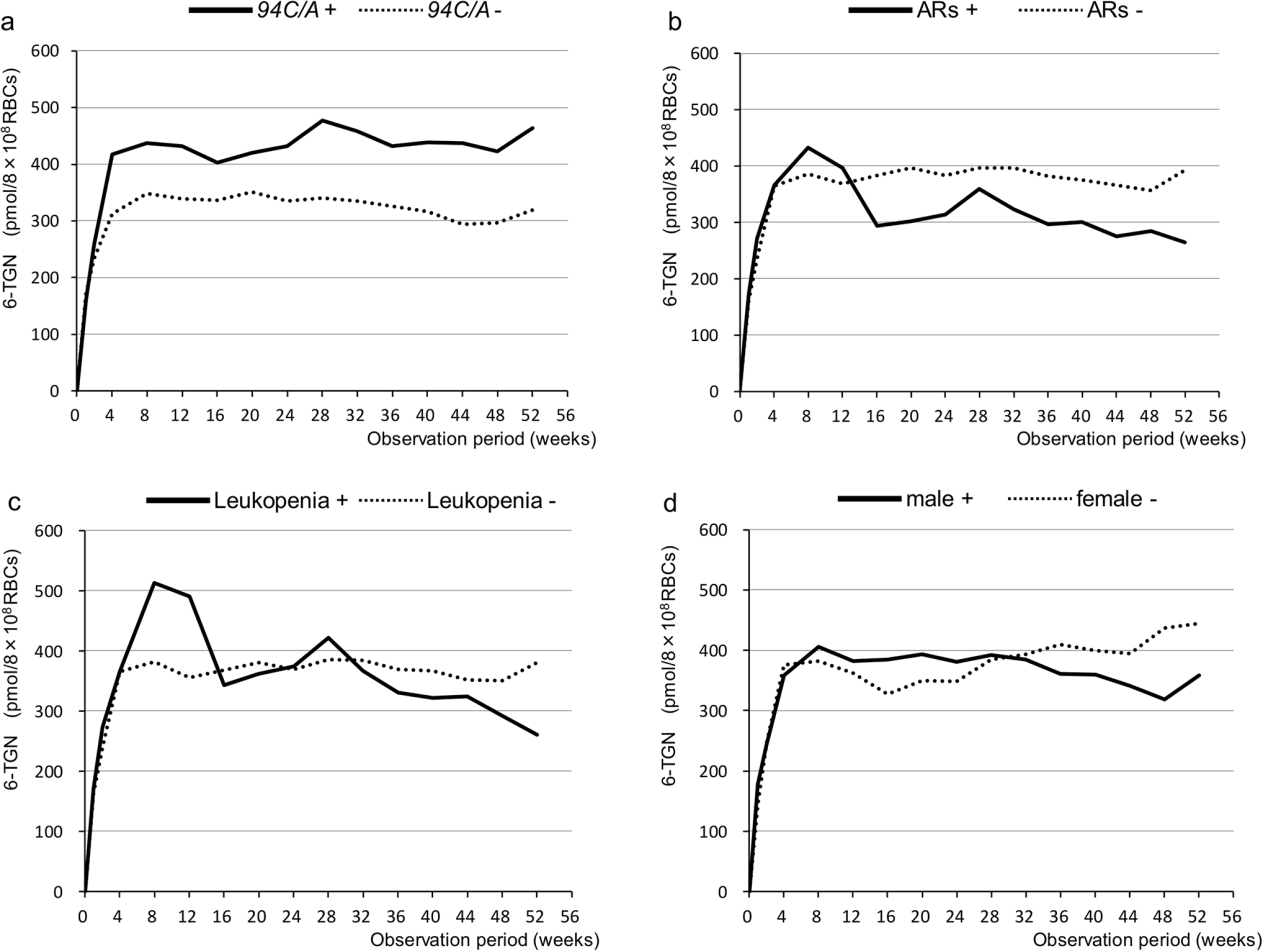
Changes in 6-TGN concentrations in RBCs (pmol/8×10^8^ RBCs) over time for the various groups. **a** The average concentration of 6-TGN in the group with *94C>A* tended to be higher than that in the group without *94C>A*. However, there was no statistically significant difference between the groups. **b c d** There were no statistically significant differences between the groups with and without adverse reactions (ARs) or the groups with and without leukopenia or between the males and females.

The average concentrations of 6-TGN in the patients with *94C>A* were generally higher than those in the patients without this mutation. However, there were no statistically significant differences between the groups (Fig 2A). There were also no statistically significant differences between the groups based on adverse reactions, leukopenia, or gender (Fig 2B, 2C and 2D).

Time points and 6-TGN concentrations in the presence of adverse reactions are shown in Table 2. Only 4 out of 14 patients with adverse reactions exhibited high 6-TGN levels (28.6%), and only 3 out of 10 patients with leukopenia exhibited high 6-TGN levels (30.0%) (>450 pmol/8×10^8^ RBCs, which is generally referred to as the toxic range [9]). The onset of adverse reactions occurred within 52 weeks from the first week after administration. Three patients with leukopenia and two with alopecia possessed low 6-TGN levels immediately after the administration of AZA.

We investigated the 6-TGN concentration changes leading to the onset of adverse reactions. Three patients with leukopenia exhibited low 6-TGN levels immediately after AZA administration. Patients with therapeutic 6-TGN levels (230–450 pmol/8×10^8^ RBCs [9]) developed leukopenia at 4, 12, 16, and 20 weeks after AZA administration. Three patients with high 6-TGN levels (over 450 pmol/8×10^8^ RBCs) developed leukopenia at 8–26 weeks after the detection of these elevated levels. One patient with agranulocytosis and alopecia had the *94C>A* allele; the concentration of 6-TGN in this individual at the time of onset was 111 pmol/8×10^8^ RBCs that was below the therapeutic window (230–260 pmol/8×10^8^ RBCs [9]).

### Correlations between WBC and 6-TGN

**Fig 3:**
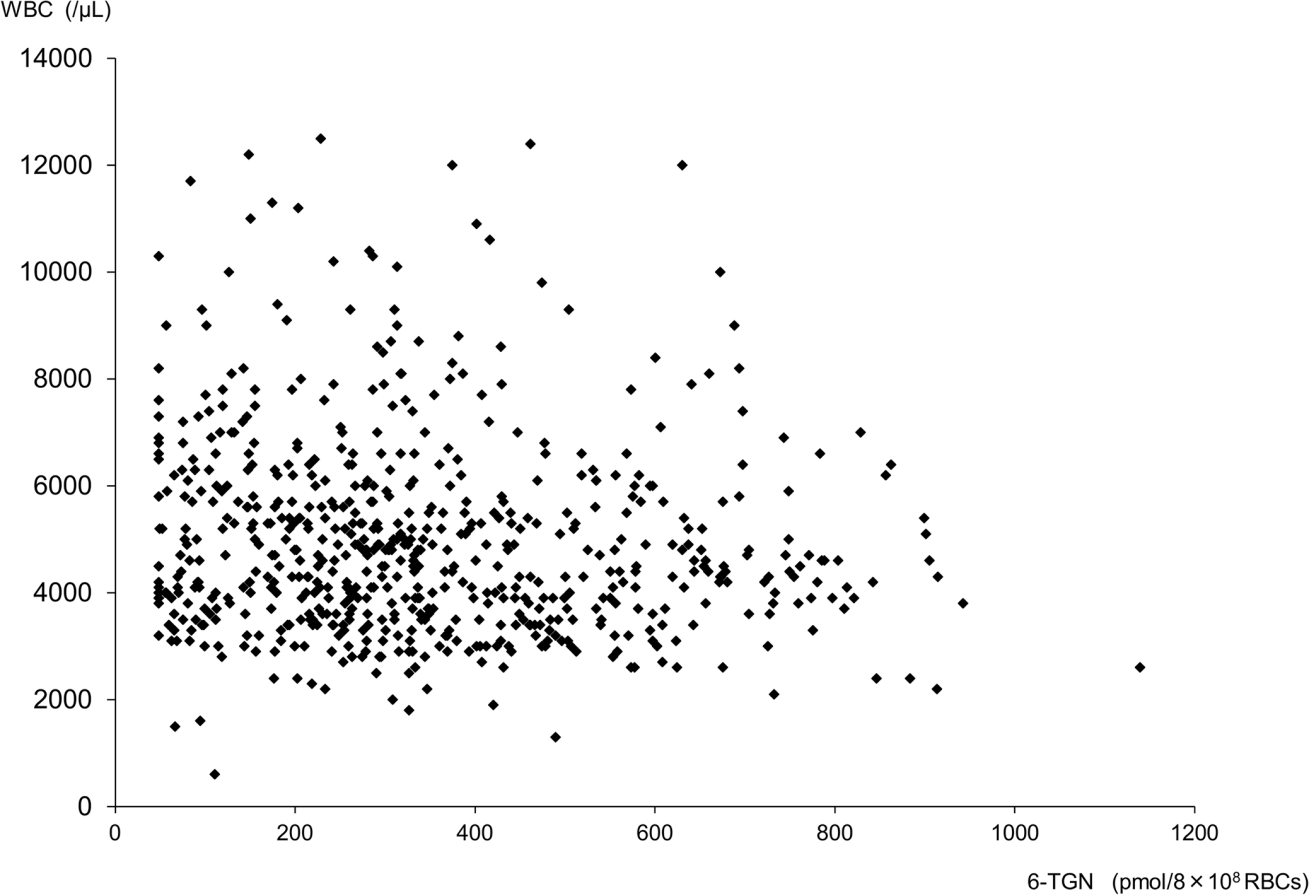
Correlations between WBC and 6-TGN. No negative correlations were observed (*r* = -0.112, *P* < 0.01, *n* = 636).

We investigated the correlations between WBC counts and 6-TGN concentrations in all samples. No negative correlations were observed (*r* = -0.112, *P* < 0.01, *n* = 636) (Fig 3).

### Cumulative incidence of leukopenia

**Fig 4:**
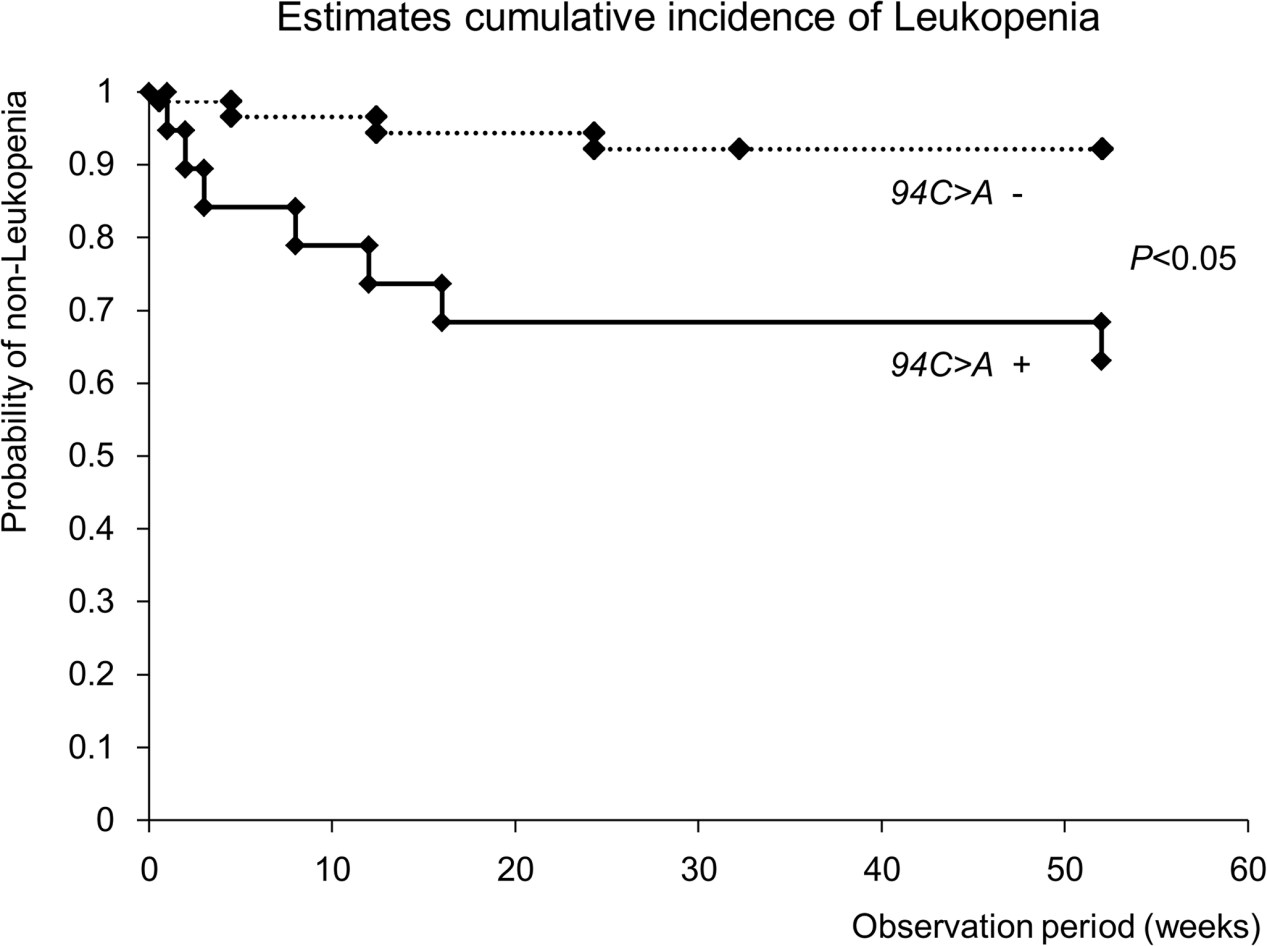
Estimates cumulative incidence of Leukopenia. Kaplan-Meier estimates show that the cumulative incidence of leukopenia was higher in the patients with *94C>A* compared with those without *94C>A*.

The Kaplan-Meier estimates of the cumulative incidence of leukopenia were higher for patients with the *94C>A* mutation compared with those without the mutation (Fig 4).

## Discussion

Previous studies indicated the main cause of AZA/6-MP-induced adverse reactions is a reduction in the activities of the metabolizing enzymes TPMT and ITPA. Influences of genetic polymorphisms on the activities of these metabolic enzymes have been reported. There are various TPMT activity-suppressing mutant alleles, and their expressions markedly vary among ethnicities. The major alleles are *2, *3A (frequently expressed in Caucasian Americans), *3B, *3C (expressed in Chinese, Ghanaians, and Japanese), *3D, *4 (found in one northern European family), *5, *6 (expressed in Koreans), *7 (expressed in Caucasian Europeans), and *8 (expressed in African Americans), and alleles up to *34 have been reported to date [5]. TPMT *3C is the major mutation observed in Japanese individuals, and reports of other mutant genes are extremely rare. Japanese patients are typically heterozygous for this mutation (TPMT *1/*3C), and it is present at a low frequency of 0.008–0.016 (0.8~1.6%) [10–12]. TPMT enzyme activity in individuals with the TPMT *1/*3C genotype is approximately 25% lower than that in wild-type individuals [13].

In addition several factors, such as ethnicity and drug interactions, play roles in TPMT activity. TPMT activity levels vary among ethnicities. Japanese patients have been reported to possess approximately 50% and 70% lower levels than those observed in Jewish and Caucasian Americans individuals, respectively [13,14,15]. According to previous reports, the concomitant use of aminosalicylic acids (ASAs), such as 5-ASA and sulfasalazine (SASP), results in significantly elevated 6-TGN levels and increases the incidence of adverse reactions, such as leukopenia [1,16,17]. Allopurinol potently inhibits XO, and a dose reduction to 25–33% of the standard daily dosage of AZA or of 6-MP is recommended when these two drugs are combined to prevent severe myelotoxicity [18]. In this study, all patients with leukopenia had been taking 5-ASA, and no patients took allopurinol.

Thiopurine-induced myelosuppression has been reported to occur dose-dependent [1]. Dubinsky *et al* noted leukopenic patients had higher 6-TGN levels [19]. A large number of reports have suggested that monitoring of erythrocyte 6-TGN concentrations may allow for adjustments in drug dose to reduce the incidence of thiopurine-induced adverse reactions in patients receiving these agents [8,19] since Cuffari *et al*. reported the existence of a correlation between 6-TGN erythrocyte levels and the clinical efficacy of 6-MP [20]. However, a few reports have shown that leukopenia could be observed with variable 6-TGN levels [21]. Ohtsuka *et al* did not report any association between 6-TGN levels and haematological toxicity [22]. In the present study, the incidences of adverse reactions did not differ between the high-level 6-TGN group (>450 pmol/8×10^8^ RBCs) and the low-level 6-TGN group (<450 pmol/8×10^8^ RBCs). Similar results were also obtained with regard to the incidence of leukopenia. Only 3 out of 10 patients with leukopenia exhibited 6-TGN concentrations exceeding 450 pmol/8×10^8^ RBCs at the time of leukopenia development. Some patients presented with sustained high 6-TGN concentrations and did not develop myelotoxicity. In the patient with agranulocytosis and alopecia, the 6-TGN concentration was 111 pmol/8×10^8^ RBCs at the time of onset. These results suggest that myelotoxicity may also occur at low 6-TGN levels, and the monitoring of 6-TGN concentrations is insufficient to predict adverse reactions. No negative correlation was noted between WBC counts and 6-TGN concentrations. Therefore, 6-TGN concentrations may not be associated with myelosuppression in Japanese IBD patients.

Several studies have reported a correlation between TPMT activity/genotype and the risk of myelotoxicity [19,23–26]. However, we did not find an association between adverse reactions and the TPMT gene mutation in the present study. Takatsu *et al*. reported that the determination of the TPMT genotype may not be useful for predicting adverse reactions to AZA in Japanese IBD patients [27]. Ban *et al*. reported that TPMT mutations are not associated with myelosuppression in Japanese IBD patients [28]. In Japanese IBD patients, myelosuppression is not considered to be solely dependent upon TPMT activity, it is also associated with other factors.

Recent studies have shown that ITPA polymorphisms are significantly associated with flu-like symptoms, rash, pancreatitis and leukopenia [29,30]. ITPA enzyme activity in RBCs is reportedly absent in individuals who are homozygous for *94C>A*, and levels are markedly lower (approximately 27% of the activity of the wild type) in those with heterozygous mutations [31,32]. The allele frequency of the *94C>A* polymorphism has been reported to be 31 out of 200 (0.155; 95% CI, 0.111–0.212) in 100 Japanese individuals [31]. This frequency is 2.6-fold higher than that reported in Caucasians (0.06) [32], indicating that many more Japanese individuals may possess the ITPA mutation compared with the TPMT mutation. It is assumed that when ITPA activity is reduced or defective, toxic 6-TITP levels accumulate and induce adverse reactions. In Japanese individuals, the ITPA gene polymorphism may be more influential compared with the TPMT polymorphism in inducing thiopurine-induced myelosuppression.

This study is the first to continuously monitor blood concentrations of 6-TGN over a long-term period in IBD patients treated with AZA. Although no significant differences were observed, the average concentrations of 6-TGN in the patients with *94C>A* were consistently higher than those in the patients without this mutation over the course of the study. Therefore, this mutation has some effects on 6-TGN levels in patients treated with thiopurines. Indeed, the incidence of leukopenia in the 19 patients with *94C>A* was significantly higher than that in the 29 patients without *94C>A* (*P* < 0.05). In addition, based on the Kaplan-Meier estimates, the cumulative incidence of leukopenia was higher for the patients with the *94C>A* mutation compared with those without it. However, only 7 of the 19 patients (36.8%) with *94C>A* developed leukopenia. Thus, this mutation does not ensure the development of adverse reactions to thiopurines, but it may represent a risk factor for leukopenia. Furthermore, factors other than increased serum levels of 6-TGN have been suggested to be involved in the onset of thiopurine-induced leukopenia in Japanese IBD patients. Further investigations are required, including the measurements of other metabolites of thiopurines, such as 6-TITP, and gene mutations in the transporter, which would affect the intracellular concentrations of important molecules, such as multidrug resistance-associated protein 4 (MRP4) [33].

In conclusion, TPMT polymorphisms did not cause adverse AZA reactions in the Japanese subjects in this study. Seven out of 19 patients (36.8%) with the ITPA *94C>A* mutation developed leukopenia; however, this mutation may not unequivocally increase the risk of developing adverse reactions to thiopurines. In addition, there are factors other than increased 6-TGN levels that are involved in the onset of leukopenia.
